# Home-Based Testing and COVID-19 Isolation Recommendations, United States

**DOI:** 10.3201/eid2909.230494

**Published:** 2023-09

**Authors:** Patrick K. Moonan, Jonathan P. Smith, Brian F. Borah, Divya Vohra, Holly H. Matulewicz, Nickolas DeLuca, Elise Caruso, Penny S. Loosier, Phoebe Thorpe, Melanie M. Taylor, John E. Oeltmann

**Affiliations:** Centers for Disease Control and Prevention, Atlanta, Georgia, USA (P.K. Moonan, B.F. Borah, N. DeLuca, E. Caruso, P.S. Loosier, P. Thorpe, M.M. Taylor, J.E. Oeltmann);; Yale University, New Haven, Connecticut, USA (J.P. Smith);; Vermont Department of Health, Burlington, Vermont, USA (B.F. Borah);; Mathematica, Princeton, New Jersey, USA (D. Vohra, H.H. Matulewicz)

**Keywords:** COVID-19, SARS-CoV-2, severe acute respiratory syndrome coronavirus 2, viruses, respiratory infections, zoonoses, isolation, diagnostics, self-testing, contact tracing, United States

## Abstract

Using a nationally representative panel survey, we examined isolation behaviors among persons in the United States who had positive SARS-CoV-2 test results during January 2021–March 2022. Compared with persons who received provider-administered results, persons with home-based results had 29% (95% CI 5%–47%) lower odds of following isolation recommendations.

Self-administered home-based tests are increasingly used as the primary method to detect SARS-CoV-2, the virus that causes COVID-19 (*1*). In contrast to tests performed at a public health department, laboratory, or other healthcare setting and administered by a provider, home-based tests require little or no interaction with the healthcare system (*2*,*3*). The Centers for Disease Control and Prevention (CDC) recommends isolation for persons who test positive for SARS-CoV-2 (*4*); however, it is unclear if test administration type is associated with following isolation recommendations. We used data from a nationally representative survey of persons in the United States with COVID-19 (*5*) to explore differences in proportions among those who isolated, followed contemporary isolation recommendations, and self-notified contacts by test administration type.

## The Study

We conducted a probability-based, web-based panel survey that provided a representative sampling frame, weighted to demographically represent all noninstitutionalized adults >18 years of age residing in the United States during January 2020–March 2022 (Appendix). For persons with multiple SARS-CoV-2 test results, isolation behaviors and self-notification of contacts corresponded to the first episode only. Because home tests were approved in late 2020 (*6*) and the recommended length of isolation duration evolved over time, we restricted survey respondents to persons with COVID-19 diagnoses that occurred during January 1, 2021–March 31, 2022, and categorized participants by whether they achieved the minimum number of days recommended for isolation on the basis of CDC-recommended contemporary isolation policies. During January 1–December 31, 2021, the minimum recommended isolation period was 10 days; during January 1–March 31, 2022 (the end date of the survey), the minimum recommended isolation period was 5 days (*7*).

We developed survey-weighted multivariable logistic models to examine the association between test administration type and 1) any isolation, 2) adherence to contemporary guidelines among those who isolated, and 3) self-reporting to contacts. We also developed a survey-weighted multivariable linear regression model to examine the association between test administration type and days of isolation. In multivariable models we controlled for age, sex, race/ethnicity, US state of residence, household size, household income, and urbanicity (i.e., urban, suburban, and rural). We transformed logistic models to compute adjusted odds ratios (aORs) and accompanying 95% CI, considering CIs that did not contain the null to be statistically significant.

Using population-weighted survey responses, we estimated 48,518,190 adults in the United States had >1 positive SARS-CoV-2 test result during the 15-month analytic period. Among those, 11,468,111 (24%) adults had results exclusively from home-based tests and 37,050,079 (76%) had results exclusively from provider-administered tests.

After we adjusted for potential confounders, persons who received results from home-based tests were significantly less likely to isolate for any duration compared with those who received provider-administered tests (78% vs. 84%; aOR 0.72 [95% CI 0.57–0.89]) (Figure). Similarly, among those who did isolate, the odds that their isolation met contemporary guidelines were significantly lower among persons who received results from home-based tests than among those with provider-administered tests (64% vs, 73%; aOR 0.71 [95% CI 0.53–0.95]). The adjusted mean duration of isolation was 2 (95% CI 1.59–2.45) days shorter among persons with results from home-based tests than those with provider-administered tests (p<0.001). Participants who home tested also had decreased odds of self-notifying their contacts; however, that association was not statistically significant (78% vs. 84%; aOR 0.79 [95% CI 0.53–1.18]) (Figure).

**Figure:**
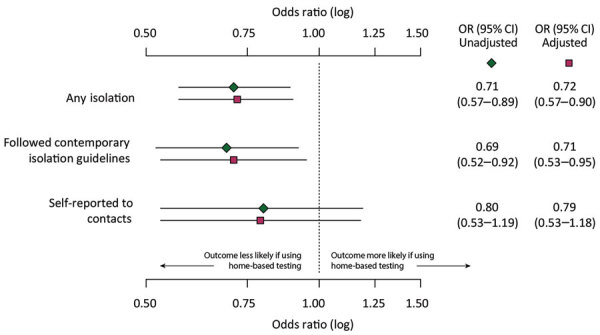
Crude and adjusted odds ratios and 95% CIs comparing COVID-19 isolation, isolation duration, and self-notification of contacts by SARS-CoV-2 test administration type, United States, January 2021–March 2022. Multivariable models included population-weighted individual survey responses controlled for age, sex, race/ethnicity, US state of residence, household size, household income, and urbanicity (i.e., urban, suburban, or rural). Isolation and notification likelihood of home-based testing is in comparison to provider-administered tests. Vertical dashed line indicates the null or no statistical association. OR, odds ratio.

## Conclusions

Using a nationally representative survey of persons with COVID-19, we found that persons in the United States who exclusively used SARS-CoV-2 home-based tests were significantly less likely to isolate or follow contemporary isolation recommendations and, on average, isolated for fewer days than those who exclusively used provider-administered tests. This analysis adds to a limited number of reports that investigated the actual behaviors of persons after they received a positive SARS-CoV-2 result. A randomized trial by Woloshin et al. (*8*) demonstrated that persons who used home-based tests might not follow CDC guidelines. Those findings suggest that persons who test at home may be unaware of or misinformed about the need for, or duration of, recommended isolation and indicates that health providers may potentially influence isolation behaviors and reinforce contemporary recommendations. Ritchey et al. (*9*) found that, despite the increased availability of home-based tests, only a small fraction of persons in the United States self-reported home-based test results to a public health surveillance system. Those findings have potential implications for initiating important public health activities, such as formal case investigation for surveillance and contract tracing to interrupt ongoing transmission. Oeltmann et al. (*5*) reported that most persons with any positive test results self-notified contacts irrespective of whether they participated in formal case investigation and contact tracing. In addition, Bien-Gund et al. found that persons who tested positive were motivated to distribute test kits to potential contacts (*10*), suggesting that persons with positive results might engage in constructive health behaviors without formal public health interactions.

The first limitation of our study is that responses were self-reported, meaning those who agreed to participate in the survey might be more health conscious and, thus, have a higher propensity to follow public health guidelines. We did not include those too ill to respond (e.g., hospitalized persons) or persons experiencing homelessness, and we only administered the survey to participants proficient in English or Spanish. Conversely, persons with mild or asymptomatic disease were plausibly less motivated to test and, thus, may have been unaware of a potential COVID-19 diagnosis, resulting in a potential misclassification in the survey. The pace of home-based testing availability and use in the study population might not reflect the true practice in the United States over time. Finally, the survey was limited to questions describing the first episode of COVID-19. For persons with multiple episodes or test results, isolation behaviors and self-notification of contacts might have changed over time.

Rapid, home-based tests for SARS-CoV-2 have both individual and public health benefits (*9*). Home-based tests greatly expanded access to COVID-19 diagnosis, especially among those without primary healthcare providers and those without stable medical benefits. However, although home-based tests increase convenience and may hasten the time to diagnosis (*2*–*4*), home-based tests eliminate the opportunity for providers to offer health education, reinforce complex and often rapidly evolving COVID-19 recommendations, and emphasize the importance of behavior change to mitigate ongoing transmission. Clear public health messaging about when and how to test, and the efficacy of each type of test, may help to ensure that persons are testing at the appropriate time, even if they do not experience any symptoms (*11*).

In our study, a notable proportion of persons with home-based test results (64%) and provider-administered test results (73%) followed contemporary isolation recommendations. Because the proportion of individuals using home-based tests has increased over time, there is a need to better integrate these results into tangible public health actions. Developing mechanisms that encourage self-report of positive home-based tests results to health departments will likely improve COVID-19 surveillance, formal case investigation, and contact tracing efforts, but also offer opportunities for additional clinical, educational, and emotional support that may further reinforce contemporary COVID-19 recommendations. Examining specific individual-level or community-level behavioral factors associated with self-reporting and other public health actions may extend these findings and deepen our understanding of optimal strategies to mitigate future pandemics with rapid widespread transmission.

Appendix Additional information about home-based COVID testing and isolation, United States, 2021–2022.
