# Supplementary material for: Home-Based Testing and COVID-19 Isolation Recommendations, United States
Source: Emerg Infect Dis. 2023 Sep;29(9):1921–4. doi: 10.3201/eid2909.230494 (PMC10461662; doi:10.3201/eid2909.230494)
Supplement: Appendix — Additional information about home-based COVID testing and isolation, United States, 2021–2022. [file 23-0494-Techapp-s1.pdf]

Article DOI: http://doi.org/10.3201/eid2909.230494

Home-Based Testing and COVID-19 Isolation Recommendations, United States

Appendix

Methods

Ipsos KnowledgePanel

We drew the sample from the Ipsos KnowledgePanel, a probability-based, web-based panel that provides a representative sampling frame for all noninstitutionalized adults (aged 18 years and older) residing in the United States (*1*). Ipsos uses an address-based sampling (ABS) recruitment method based on the U.S. Postal Service's Delivery Sequence File. ABS may improve population coverage, and also provides a more effective means for recruiting hard-to-reach individuals, such as cellphone-only households, non-internet households, young adults, and persons of color. Households without an internet connection are provided with a web-enabled device and free internet service.

For this study, a sample of 22,514 panelists were selected and invited to complete the survey. A total of 15,923 responded to the survey invitation and completed the survey, resulting in a study completion rate of 71%. In total, 3,500 respondents self-reported a positive SARS-CoV-2 test result (cases), 5,369 respondents self-reported exposure to a person with COVID-19 (contacts) and 6,654 were neither a case nor a contact (controls) (*2*).

Sampling and Administration

Stratified random sampling and weighing ensures that the geodemographic composition is comparable with that of the adult U.S. population (Supplement Table). Adults from sampled households are invited to join KnowledgePanel through ABS using a series of mailings, including an initial invitation letter, a reminder postcard, and a subsequent follow-up letter. Moreover, telephone refusal-conversion calls are made to nonresponding households for which a telephone number could be matched to a physical address.

To increase the representativeness of U.S. Hispanics in KnowledgePanel, Hispanic members recruited through Ipsos' traditional ABS sampling methodology described above are

supplemented with recruitment using a custom dual-frame random-digit dialing sampling methodology targeting telephone exchanges associated with census blocks that have a 65% or greater Latino population density of the U.S. Hispanic population. Moreover, cellular numbers from rates centers with high concentration of Hispanics are also used to improve the representation of samples. With this telephone recruitment, households are screened in the Spanish language to only recruit those homes where Spanish is spoken at least half the time.

Once panel members are recruited and profiled by completing the Core Profile Survey, they are considered "active members" and become eligible for selection for specific surveys. Typically, specific survey samples are based on an equal probability selection method (EPSEM) for general population surveys. For selection of general population samples from KnowledgePanel, a patented methodology has been developed such that samples from the panel behave as EPSEM samples. Briefly, this methodology starts by weighting the pool of active members to the geodemographic benchmarks secured from a combination of the U.S. Census Bureau's American Community Survey (ACS) and the latest March supplement of the U.S. Census Bureau's Current Population Survey (CPS) along several dimensions. Using the resulting weights as measures of size (calculated at the person level), a probability-proportional-to-size (PPS) procedure is used to select study specific samples. The primary sampling unit is the individual person. It is the application of this PPS methodology with the imposed size measures that produces demographically balanced and representative samples that behave as EPSEM. Once assigned to a survey, members receive a notification email letting them know there is a new survey available for them to complete. Typically, after 3 days, automatic email reminders are sent to all non-responding panel members in the sample. Additional email reminders are sent and custom reminder schedules are set up as needed.

Study-Specific Weights

For this study, our weighting process included the following steps:

1. In the first step, design weights for all KnowledgePanel (KP) assignees were computed to reflect their selection probabilities.
2. The above design weights for KP respondents, regardless of qualification status to our survey, were adjusted to represent all persons aged 18 years and over in the U.S. population for the following geodemographic variables and categories using an iterative proportional fitting (raking) procedure. The needed benchmarks were

obtained from the 2021 March Supplement of the Current Population Survey, except language proficiency, which is not available, was obtained from the 2019 American Community Survey. Because race/ethnicity is an important analytical variable, we included some adjustments within race/ethnicity categories. Samples sizes were sufficient to support these nested adjustments (Supplement Table).

a. Age (18–29, 30–44, 45–59, 60+) by Gender (Male, Female) by Race-Ethnicity (Non-Hispanic White, Non-Hispanic Black, Non-Hispanic Other/2+ Races, Hispanic)

b. Education (Less than High School, High School, Some College, Bachelor or Higher) by Race-Ethnicity (Non-Hispanic White, Non-Hispanic Black, Non-Hispanic Other/2+ Races, Hispanic)

c. Household Income (Under $25,000 $25,000–$49,999, $50,000–$74,999, $75,000–$99,999, $100,000–$149,999, $150,000 and over) by Race-Ethnicity (Non-Hispanic White, Non-Hispanic Black, Non-Hispanic Other/2+ Races, Hispanic)

d. Census Region (Northeast, Midwest, South, and West) by Race-Ethnicity (Non-Hispanic White, Non-Hispanic Black, Non-Hispanic Other/2+ Races, Hispanic)

e. Metropolitan Status (Metro, Non-Metro) by Race-Ethnicity (Non-Hispanic White, Non-Hispanic Black, Non-Hispanic Other/2+ Races, Hispanic)

f. Race-Ethnicity (Non-Hispanic White, Non-Hispanic Black, Non-Hispanic American Indian or Alaska Native, Non-Hispanic Asian, Native Hawaiian, Pacific Islander, Non-Hispanic Other Race/2+ Races, Hispanic)

g. Hispanic Origin (Non-Hispanic, Mexican Hispanic, Puerto Rican Hispanic, Cuban Hispanic, Other Hispanic Origins)

h. Language Proficiency within Hispanic (English Proficient Hispanic, Bilingual Hispanic, Spanish Proficient Hispanic, Non-Hispanic)

3. In the final step, the resulting weights were trimmed as needed and scaled to sum to the 18 and over U.S. population size.

Nonresponse Bias Analysis

In all survey-only based studies, final estimates are based solely on the survey's respondents. Errors may arise in the estimates resulting from nonresponse if there are systematic differences between persons who respond to a survey and those who do not. Nonresponse-adjusted weights attempt to account for these differences by identifying characteristics available for both respondents and nonrespondents that are associated both with the likelihood of responding and key outcomes. This is done by adjusting the weights of the respondents to compensate for the nonrespondents using these characteristics. In studies where these adjustments can successfully account for differences between nonrespondents and respondents, the survey estimates would have minimal potential for nonresponse bias.

The nonresponse adjustments applied to the sampling weights in the National Survey of Health in America, 2022 appear to have effectively accounted for differences between respondents and nonrespondents, thereby minimizing the potential for nonresponse bias. The study team cannot directly measure nonresponse bias without knowing how nonrespondents would have answered survey items; however, we can examine variables available for both respondents and nonrespondents that we believe are correlated with responses to survey items.

Our analysis indicates that the nonresponse adjustment alleviated differences observed between respondents and nonrespondents in the sample for the variables that we had at our disposal. The largest relative differences that did exist occurred with age by sex by race/ethnicity, education by race/ethnicity, income by race/ethnicity, Metropolitan Statistical Areas (MSA) by race/ethnicity, and race/ethnicity.

Results

We used the initial weights to compare the distributions of the variables across the frame and the total sample. We also compared the distributions of variables between the respondents and nonrespondents, to establish how respondents and nonrespondents differed. We then compared estimates using respondents only (using nonresponse-adjusted weights) and the Current Population Survey (CPS) estimates reported in the Supplement Table. We used SAS survey procedures to calculate standard errors to properly account for unequal weights. The sample statistics consist of proportions with an attribute (presented as percentages). The variables have trivial numbers of missing, and in each case, the proportions with each attribute

that were used in the following analyses were calculated among cases without missing data. The values are percentages for each level of the categorical variables, with the associated standard errors (*se*).

Comparison of Entire Sample with Frame

Before conducting a nonresponse analysis, the first step is to check whether the sample distribution adequately matches the frame distribution on important variables that may not have been controlled for in the sampling process. This is necessary to ascertain whether the estimates using the sampling weights produce estimates that are consistent with population values. Statistics estimated from the entire sample (using the initial sampling weight) among all adults are close to those computed with the full frame but are not exact. The sample somewhat underrepresents young, black men (18–29 Male Non-Hispanic Black) and overrepresents young, non-Hispanic women (18–29 Female Non-Hispanic White). The sample also somewhat overrepresents lower income whites (Under $24,999, Non-Hispanic White, $25,000–$49,999 Non-Hispanic White) and underrepresents high income adults regardless of race/ethnicity (income $150,000 and over) (not shown).

Assessment of Differences between Respondents and Nonrespondents before Nonresponse Adjustment

We then compared respondents and nonrespondents. We calculated the *t*-statistic by calculating the differences between the proportions within the levels of each demographic covariate and creating an estimate of the variance of the difference by combining the standard error estimates obtained from the SAS survey procedure. Respondents were different from nonrespondents for most variables we examined. The drivers of nonresponse appear to be that (1) age is very highly correlated with response, with the highest response rate among adults over 60, and (2) response among blacks and Hispanics is low relative to all other groups. Respondents were also more likely to be higher educated (bachelor's degree or higher) (not shown).

Nonresponse Adjustment

Nonresponse adjustments made to initial weights seek to reduce the potential for bias that might result from differential nonresponse based on a set of variables. These variables should be available for both respondents and nonrespondents, be related to the likelihood of responding, and be correlated with key survey outcomes. The sampling design weights for survey respondents —including all who answered the screener questions regardless of case or contact status — were adjusted to represent all adults in the U.S. population for the geodemographic

variables and categories using an iterative proportional fitting (raking) procedure. The needed benchmarks were obtained from the 2021 March Supplement of the CPS, except language proficiency, which is not available from CPS and was obtained from the 2019 American Community Survey.

Age (18–29, 30–44, 45–59, 60+) by Gender (Male, Female) by Race-Ethnicity (Non-Hispanic White, Non-Hispanic Black, Non-Hispanic Other/2+ Races, Hispanic)

Education (Less than High School, High School, Some College, Bachelor or Higher) by Race-Ethnicity (Non-Hispanic White, Non-Hispanic Black, Non-Hispanic Other/2+ Races, Hispanic)

Household Income (Under $24,999, $25,000–$49,999, $50,000–$74,999, $75,000–$99,999, $100,000–$149,999, $150,000 and over) by Race-Ethnicity (Non-Hispanic White, Non-Hispanic Black, Non-Hispanic Other/2+ Races, Hispanic)

Census Region (Northeast, Midwest, South, and West) by Race-Ethnicity (Non-Hispanic White, Non-Hispanic Black, Non-Hispanic Other/2+ Races, Hispanic)

Metropolitan Status (Metro, Non-Metro) by Race-Ethnicity (Non-Hispanic White, Non-Hispanic Black, Non-Hispanic Other/2+ Races, Hispanic)

Race-Ethnicity (Non-Hispanic White, Non-Hispanic Black, Non-Hispanic American Indian or Alaska Native, Non-Hispanic Asian, Native Hawaiian, Pacific Islander, Non-Hispanic Other Race/2+ Races, Hispanic)

Hispanic Origin (Non-Hispanic, Mexican Hispanic, Puerto Rican Hispanic, Cuban Hispanic, Other Hispanic Origins)

Language Proficiency within Hispanic (English Proficient Hispanic, Bilingual Hispanic, Spanish Proficient Hispanic, Non-Hispanic)

In the final step, the resulting weights were trimmed and scaled to sum to all adults in U.S. population census. Comparison of respondents and ineligibles to the sampling frame after nonresponse adjustment. The purpose of nonresponse adjustments is to account for any differences between respondents and nonrespondents, to make respondents look like the original sample as much as possible.

In this section, we evaluate how well the nonresponse adjustments accounted for those differences. Below, we included percentages from the CPS, estimated percentages from the entire sample (using initial sampling weights), and nonresponse-adjusted weighted estimated percentages among respondents. We compare estimates using nonresponse-adjusted weights to the CPS because the survey panel distribution should match the CPS distributions. The nonresponse adjustments to the sampling weights alleviated most of the differences observed between respondents and nonrespondents (Appendix Table). When compared to CPS there are no differences. As described above, the weights are adjusted to CPS distributions and the final nonresponse adjustments result in no differences between the weighted estimates and CPS distributions.

Summary and Implications for Analyses

Our analysis has shown that the selected sample for the National Survey of Health in America, 2022 was representative of the populations of interest among variables used for selection. Because we did not achieve an 80% response rate, the main purpose of this nonresponse bias analysis was to determine if systematic differences between respondents and nonrespondents were alleviated by nonresponse adjustments to the weights, or if the potential for nonresponse bias was still likely in the weighted estimates.

We found that the nonresponse adjustments alleviated most of the differences observed between respondents and nonrespondents. In addition, it does not appear that the nonresponse adjustments created new biases.

Appendix References

1. Ipsos. Ipsos knowledge panel. [cited 2023 Jul 20]. https://www.ipsos.com/en-us/solutions/public-affairs/knowledgepanel.

2. Oeltmann JE, Vohra D, Matulewicz HH, DeLuca N, Smith JP, Couzens C, et al. Isolation and quarantine for COVID-19 in the United States, 2020–2022. Clin Infect Dis. 2023. [Epub ahead of print.] https://doi.org/10.1093/cid/ciad163

Appendix Table. Percentages and standard errors of various demographic attributes comparing Current Population Survey estimates with final weighted estimate using nonresponse-adjusted weights

Variable	CPS percent	Entire sample percent using initial weights: Percent	*se*	Number of respondents with attribute	Respondent-based weighted percent using nonresponse-adjusted weights: Percent	*se*
Age, y, Sex, Race/Ethnicity						
18–29 Male, Non-Hispanic White	5.5	5.4	0.16	601	5.5	0.22
18–29 Female, Non-Hispanic White	5.3	6.9	0.18	734	5.3	0.20
30–44 Male, Non-Hispanic White	7.2	7.4	0.18	1139	7.3	0.21
30–44 Female, Non-Hispanic White	7.2	6.7	0.18	977	7.2	0.23
45–59 Male, Non-Hispanic White	7.3	7.4	0.18	1358	7.3	0.20
45–59 Female, Non-Hispanic White	7.6	6.8	0.17	1200	7.6	0.22
60+ Male, Non-Hispanic White	10.5	11.0	0.21	2346	10.5	0.22
60+ Female, Non-Hispanic White	11.9	11.0	0.21	2210	12.0	0.25
18–29 Male, Non-Hispanic Black	1.4	0.8	0.06	80	1.3	0.16
18–29 Female, Non-Hispanic Black	1.4	1.4	0.08	112	1.4	0.15
30–44 Male, Non-Hispanic Black	1.5	1.3	0.08	197	1.6	0.12
30–44 Female, Non-Hispanic Black	1.7	2.0	0.09	260	1.8	0.11
45–59 Male, Non-Hispanic Black	1.3	1.4	0.07	249	1.3	0.09
45–59 Female, Non-Hispanic Black	1.6	1.7	0.08	306	1.6	0.10
60+ Male Non-Hispanic Black	1.3	1.5	0.08	314	1.3	0.08
60+ Female Non-Hispanic Black	1.8	1.9	0.09	401	1.8	0.10
18–29 Male, Non-Hispanic Other/2+ Races	1.0	0.8	0.07	76	1.0	0.13
18–29 Female, Non-Hispanic Other/2+ Races	1.0	1.0	0.07	82	0.9	0.13
30–44 Male Non-Hispanic Other/2+ Races	1.2	1.3	0.08	192	1.2	0.11
30–44 Female, Non-Hispanic Other/2+ Races	1.4	1.5	0.08	205	1.4	0.13
45–59 Male, Non-Hispanic Other/2+ Races	0.9	1.2	0.07	189	0.9	0.09
45–59 Female, Non-Hispanic Other/2+ Races	1.1	1.0	0.07	173	1.1	0.11
60+ Male Non-Hispanic Other/2+ Races	0.8	0.9	0.06	177	0.8	0.09
60+ Female Non-Hispanic Other/2+ Races	1.1	1.0	0.07	186	1.1	0.12
18–29 Male, Hispanic	2.4	1.8	0.09	162	2.3	0.21
18–29 Female, Hispanic	2.3	2.3	0.10	232	2.3	0.16
30–44 Male, Hispanic	2.7	2.6	0.11	363	2.7	0.16
30–44 Female, Hispanic	2.6	2.8	0.11	332	2.6	0.16
45–59 Male, Hispanic	2.0	2.2	0.10	371	2.0	0.12
45–59 Female, Hispanic	2.0	2.2	0.10	360	2.0	0.12
60+ Male, Hispanic	1.3	1.6	0.08	327	1.3	0.08
60+ Female, Hispanic	1.6	1.4	0.08	256	1.6	0.11
Education, Race/Ethnicity						
Less than High School, Non-Hispanic White	3.6	4.3	0.16	469	3.6	0.17
High School, Non-Hispanic White	16.9	17.7	0.28	2434	17.0	0.33
Some College, Non-Hispanic White	17.4	16.8	0.25	2971	17.4	0.31
Bachelor or Higher, Non-Hispanic White	24.6	23.8	0.28	4691	24.7	0.34
Less than High School, Non-Hispanic Black	1.2	1.1	0.08	123	1.2	0.12
High School, Non-Hispanic Black	4.1	3.6	0.13	460	4.1	0.22
Some College, Non-Hispanic Black	3.6	3.9	0.12	667	3.6	0.16
Bachelor or Higher, Non-Hispanic Black	3.1	3.4	0.11	669	3.1	0.13
Less than High School, Non-Hispanic Other/2+ Races	0.7	0.5	0.05	41	0.6	0.12
High School, Non-Hispanic Other/2+ Races	1.8	1.6	0.10	172	1.7	0.16
Some College, Non-Hispanic Other/2+ Races	1.9	2.1	0.10	299	1.9	0.16
Bachelor or Higher, Non-Hispanic Other/2+ Races	4.2	4.5	0.14	768	4.3	0.19
Less than High School, Hispanic	4.1	3.7	0.14	406	4.1	0.23
High School, Hispanic	5.5	5.4	0.16	645	5.5	0.24
Some College, Hispanic	4.2	4.3	0.13	716	4.2	0.18
Bachelor or Higher, Hispanic	3.1	3.4	0.11	636	3.1	0.14
Income, Race/Ethnicity						
Under $24,999, Non-Hispanic White	6.9	8.4	0.20	1135	6.9	0.21
$25,000–$49,999, Non-Hispanic White	9.7	10.6	0.21	1674	9.8	0.24
$50,000–$74,999, Non-Hispanic White	9.7	10.1	0.21	1643	9.7	0.24
$75,000–$99,999, Non-Hispanic White	8.4	8.8	0.19	1518	8.4	0.22
$100,000–$149,999, Non-Hispanic White	11.9	11.4	0.21	2033	11.9	0.26
$150,000 or more, Non-Hispanic White	16.0	13.2	0.22	2562	16.1	0.31
Under $24,999, Non-Hispanic Black	2.6	3.4	0.12	429	2.6	0.15
$25,000–$49,999 Non-Hispanic Black	2.5	2.5	0.10	396	2.5	0.15
$50,000–$74,999 Non-Hispanic Black	2.2	2.1	0.09	351	2.2	0.15
$75,000–$99,999 Non-Hispanic Black	1.4	1.4	0.07	264	1.4	0.10
$100,000–$149,999 Non-Hispanic Black	1.7	1.5	0.08	274	1.7	0.12
$150,000 or more Non-Hispanic Black	1.6	1.1	0.07	205	1.6	0.13
Under $24,999, Non-Hispanic Other/2+ Races	0.9	1.2	0.08	132	0.9	0.11

Variable	CPS percent	Entire sample percent using initial weights: Percent	*se*	Number of respondents with attribute	Respondent-based weighted percent using nonresponse-adjusted weights: Percent	*se*
$25,000–$49,999, Non-Hispanic Other/2+ Races	1.1	1.3	0.08	174	1.1	0.12
$50,000–$74,999, Non-Hispanic Other/2+ Races	1.2	1.4	0.08	202	1.2	0.12
$75,000–$99,999, Non-Hispanic Other/2+ Races	1.0	1.2	0.08	200	1.0	0.11
$100,000–$149,999, Non-Hispanic Other/2+ Races	1.6	1.5	0.08	256	1.5	0.12
$150,000 and more, Non-Hispanic Other/2+ Races	2.8	1.9	0.09	316	2.7	0.18
Under $24,999, Hispanic	2.4	3.7	0.13	401	2.4	0.14
$25,000–$49,999, Hispanic	3.7	4.4	0.14	560	3.7	0.18
$50,000–$74,999, Hispanic	3.2	3.1	0.12	456	3.2	0.18
$75,000–$99,999, Hispanic	2.4	2.0	0.09	314	2.4	0.16
$100,000–$149,999, Hispanic	2.8	2.1	0.09	387	2.8	0.16
$150,000–Over Hispanic	2.4	1.6	0.08	285	2.4	0.17
Geographic Region, Race/Ethnicity						
Northeast, Non-Hispanic White	11.6	12.0	0.23	1959	11.7	0.26
Midwest, Non-Hispanic White	16.1	16.0	0.25	2794	16.1	0.30
South, Non-Hispanic White	22.3	22.0	0.28	3617	22.3	0.35
West, Non-Hispanic White	12.6	12.6	0.23	2195	12.6	0.27
Northeast, Non-Hispanic Black	1.8	1.8	0.09	280	1.8	0.13
Midwest, Non-Hispanic Black	2.0	2.1	0.09	336	2.0	0.13
South, Non-Hispanic Black	7.0	6.9	0.17	1117	7.0	0.24
West, Non-Hispanic Black	1.1	1.1	0.07	186	1.1	0.10
Northeast, Non-Hispanic Other/2+ Races	1.5	1.4	0.08	196	1.5	0.14
Midwest, Non-Hispanic Other/2+ Races	1.1	1.3	0.08	202	1.1	0.11
South, Non-Hispanic Other/2+ Races	2.4	2.6	0.11	379	2.3	0.16
West, Non-Hispanic Other/2+ Races	3.6	3.3	0.12	503	3.5	0.20
Northeast, Hispanic	2.2	2.0	0.09	287	2.2	0.15
Midwest, Hispanic	1.4	1.3	0.07	192	1.4	0.12
South, Hispanic	6.6	6.7	0.16	956	6.6	0.25
West, Hispanic	6.6	6.9	0.17	968	6.6	0.24
MSA Category, Race/Ethnicity						
Non-Metro, Non-Hispanic White	10.8	11.2	0.22	1732	10.8	0.26
Metro, Non-Hispanic White	51.8	51.4	0.34	8833	51.9	0.45
Non-Metro, Non-Hispanic Black	1.0	1.0	0.07	143	1.0	0.10
Metro, Non-Hispanic Black	11.0	11.0	0.20	1776	11.0	0.29
Non-Metro, Non-Hispanic Other/2+ Races	0.7	0.8	0.06	118	0.7	0.09
Metro, Non-Hispanic Other/2+ Races	8.0	7.8	0.19	1162	7.8	0.29
Non-Metro, Hispanic	1.0	0.9	0.07	130	1.0	0.10
Metro, Hispanic	15.9	15.9	0.24	2273	15.9	0.36
Race/Ethnicity						
Non-Hispanic White	62.5	62.5	0.33	10565	62.7	0.45
Non-Hispanic Black	12.0	12.0	0.21	1919	12.0	0.31
Non-Hispanic Asian, Native Hawaiian, Pacific Islander	6.4	4.0	0.13	628	6.3	0.28
Hispanic	16.9	16.9	0.25	2403	16.9	0.37
Non-Hispanic Other Race/2+ Races	2.2	4.6	0.15	652	2.2	0.13
Hispanic/Latino Origin						
Non-Hispanic	83.1	83.1	0.25	13764	83.1	0.37
Mexican, Hispanic	10.2	10.2	0.20	1397	10.2	0.30
Puerto Rican, Hispanic	1.5	1.6	0.08	246	1.5	0.11
Cuban, Hispanic	0.8	0.8	0.06	140	0.8	0.09
Other, Hispanic Origin	4.3	4.3	0.13	620	4.3	0.21

*CPS, Current Population Survey; se, standard error
